# Aerobic exercise and aerobic fitness level do not modify motor learning

**DOI:** 10.1038/s41598-021-84764-y

**Published:** 2021-03-08

**Authors:** Andrea Hung, Marc Roig, Jenna B. Gillen, Catherine M. Sabiston, Walter Swardfager, Joyce L. Chen

**Affiliations:** 1 Faculty of Kinesiology and Physical Education, University of Toronto, 55 Harbord Street, Toronto, ON M5S 2W6 Canada; 2 Rehabilitation Sciences Institute, University of Toronto, Toronto, ON Canada; 3 Memory and Motor Rehabilitation Laboratory (MEMORY-LAB), Feil and Oberfeld Research Centre, Jewish Rehabilitation Hospital, Montreal Center for Interdisciplinary Research in Rehabilitation (CRIR), Laval, QC Canada; 4 School of Physical and Occupational Therapy, McGill University, Montreal, QC Canada; 5 Hurvitz Brain Sciences Program, Sunnybrook Research Institute, Toronto, ON Canada; 6 Department of Pharmacology and Toxicology, University of Toronto, Toronto, ON Canada

**Keywords:** Motor control, Human behaviour, Neuroscience, Learning and memory, Consolidation

## Abstract

Motor learning may be enhanced when a single session of aerobic exercise is performed immediately before or after motor skill practice. Most research to date has focused on aerobically trained (AT) individuals, but it is unknown if aerobically untrained (AU) individuals would equally benefit. We aimed to: (a) replicate previous studies and determine the effect of rest (REST) versus exercise (EXE) on motor skill retention, and (b) explore the effect of aerobic fitness level (AU, AT), assessed by peak oxygen uptake (VO_2_peak), on motor skill retention after exercise. Forty-four participants (20–29 years) practiced a visuomotor tracking task (acquisition), immediately followed by 25-min of high-intensity cycling or rest. Twenty-four hours after acquisition, participants completed a motor skill retention test. REST and EXE groups significantly improved motor skill performance during acquisition [*F*(3.17, 133.22) = 269.13, *P* = 0.001], but had no group differences in motor skill retention across time. AU-exercise (VO_2_peak = 31.6 ± 4.2 ml kg^−1^ min^−1^) and AT-exercise (VO_2_peak = 51.5 ± 7.6 ml kg^−1^ min^−1^) groups significantly improved motor skill performance during acquisition [*F*(3.07, 61.44) = 155.95, *P* = 0.001], but had no group differences in motor skill retention across time. Therefore, exercise or aerobic fitness level did not modify motor skill retention.

## Introduction

As a society, we are fascinated by the pursuit of pushing human performance to the next level. We are constantly seeking ways to become faster, stronger, and more powerful. In the field of motor learning, performance is commonly optimized using well-established principles like practice organization and augmented feedback^1^. However, what if strategies beyond motor learning principles could be used to yield superior performance outcomes? There is growing interest in exploring novel approaches that harness the brain’s neuroplasticity, such as non-invasive brain stimulation^2^ and aerobic exercise^3^. These approaches are believed to interact with learning-related mechanisms in the brain. As a result, applying these approaches before, during, or after motor skill practice may lead to additional enhancements in motor learning^2,3^.

Emerging research suggests a single session of aerobic exercise may enhance motor learning when the exercise is performed immediately before or after practice of a new motor skill (for neuroplasticity mechanisms, refer to later section below)^4–13^. Potential moderators that could impact the effects of exercise on motor learning include exercise intensity and exercise timing. For example, the effects may be more salient when aerobic exercise is performed at a high-intensity^13^ and with minimal time delay between the end of acquisition and beginning of exercise^11^. Furthermore, the effects of exercise on motor learning may not be limited to aerobic exercise alone. Other forms of high-intensity exercise, such as circuit training, resistance training, and sport applications may also enhance motor learning^14^.

This prior body of literature has mainly investigated individuals with high aerobic fitness, typically assessed by peak oxygen uptake (VO_2_peak) during a maximal graded exercise test. Specifically, participants’ aerobic fitness levels have predominantly been in the good to excellent range^5–8,11,12, 14,15^ (females: 39.5–46.8 ml kg^−1^ min^−1^; males: 45.6–54.0 ml kg^−1^ min^−1^; see Supplementary Table S1 online), based on the American College of Sports Medicine’s fitness categories^16^. However, individuals with high aerobic fitness only represent one extreme of the general population. It is unknown if individuals with low aerobic fitness can benefit from a single session of aerobic exercise for motor learning to the same extent as those with high aerobic fitness. Two studies have tested individuals with aerobic fitness levels in the poor to fair range^4,17^ (females: 32.3–38.5 ml kg^−1^ min^−1^; males: 38.0–44.8 ml kg^−1^ min^−1^). One study found aerobic exercise enhanced motor learning^4^. In contrast, another study did not demonstrate a statistically significant effect of aerobic exercise on motor learning, albeit 25% greater motor skill retention in the exercise group compared to control^17^.

To our knowledge, no studies have directly tested if aerobic fitness level impacts the effect of a single session of aerobic exercise on motor learning. Two studies have examined the general effect of aerobic fitness level on motor learning, but did not include a session of aerobic exercise^18,19^. One study found that aerobically fit individuals had superior motor learning than individuals who were less aerobically fit^18^. However, a confound in this study is that the aerobically fit group included younger adults (20–40 years old), while the less aerobically fit group included older adults (60–80 years old). The second study did not find aerobic fitness level affected motor learning^19^. However, a limitation of the study is that an arbitrary cut off value (i.e. median split approach) was used to categorize individuals into aerobically fit and less aerobically fit groups.

Aerobic fitness level could be an important mediator of exercise effects on motor learning because of the notion of “responders” and “non-responders” to exercise. There is variability in how much an individual can improve their aerobic fitness level^20,21^, and how one’s body physiologically adapts to exercise (e.g. lactate threshold^20^ and cardiovascular function^22^). More relevant for the present study, the effects of aerobic exercise on motor learning are believed to be related to the brain’s neuroplasticity^3^, which may be modulated by aerobic fitness level^23^. At the metabolic level, a single session of aerobic exercise results in elevated brain derived neurotrophic factor (BDNF) levels that may be associated with better motor skill retention^8^; though a second study did not find the same association^5^. Further, lower aerobic fitness levels are associated with reduced BDNF release after a single session of aerobic exercise compared with higher aerobic fitness levels^24^. At the cortical level, a single session of aerobic exercise increases corticospinal tract excitability (CSE) and reduces GABA_A_ inhibition in the motor cortex, both of which are associated with improvements in motor skill retention^10,17^. However, individuals who are inactive or have low physical activity levels do not have changes in CSE after a single session of aerobic exercise compared to those with high physical activity levels^25,26^. Therefore, individuals with different aerobic fitness levels may have variable neuroplasticity-related responses to aerobic exercise (e.g., changes in neurochemical release or CSE), and thus, have different motor learning outcomes after aerobic exercise.

The primary objective of this study is to replicate past studies and determine the effect of rest (REST) versus exercise (EXE) on motor skill retention using a between-subjects study design. We hypothesize motor skill retention would be superior after exercise compared to rest. The secondary objective of this study is novel and exploratory: to investigate the effect of aerobic fitness level (Aerobically Untrained [AU] versus Aerobically Trained [AT]) on motor skill retention after exercise using a between-subjects study design. We hypothesize motor skill retention is superior in the Aerobically Trained-exercise (AT-EXE) group compared to the Aerobically Untrained-exercise (AU-EXE) group.

## Results

**Figure 1:**
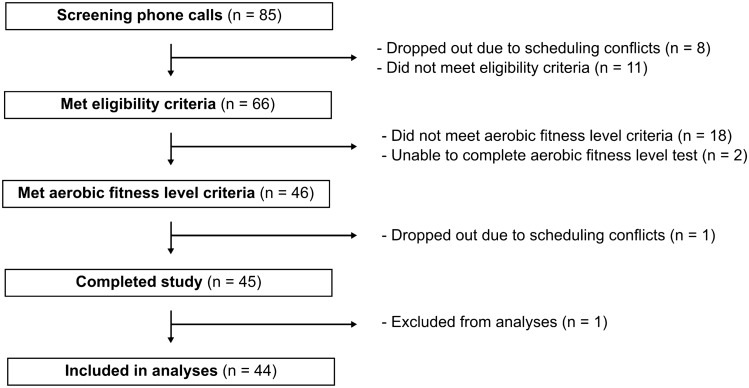
Participant flow diagram.

Participants were screened for eligibility: healthy, right-handed, young adults (20–29 years old) with no medical diagnoses, not taking medications that affect the central nervous system, and do not consume nicotine or cannabis. Participants who met the eligibility criteria were invited for aerobic fitness level screening (Visit 1). If participants met the aerobic fitness level criteria, they were invited to complete the remainder of the study (Visit 2: motor skill acquisition, followed by either rest or exercise; Visit 3: motor skill retention test). One participant in the AT-EXE group was excluded from analyses because they did not follow the directions during motor skill acquisition, despite repeated attempts by the researcher to reiterate the instructions. Refer to Fig. 1 for the flow of participants through the study.

Two sets of analyses were conducted for this study. We first compared REST and EXE groups to determine the overall effect of exercise on motor learning. Subsequently, we performed exploratory sub-analyses to compare AU-EXE and AT-EXE groups to understand the potential moderator effect of aerobic fitness level on exercise effects on motor learning.

### Demographics

During Visit 1, participants’ aerobic fitness levels were determined using a maximal graded exercise test. Aerobic fitness level was defined by VO_2_peak, where a lower value reflected a lower aerobic fitness level. Participants were assigned to the AU or AT group if their VO_2_peak was within the bottom 25% or top 25% of age- and sex-specific normative values^16^ respectively, and then pseudorandomized into either the REST or EXE group to match age and sex.

Full statistical results for demographics are presented in Table 1. Independent two-tailed t-tests were performed to compare REST and EXE groups. There were no differences in age, body mass index (BMI), handedness, VO_2_peak, or peak cycling power (Wpeak). Independent two-tailed t-tests were also performed to compare age, BMI, and handedness between AU-EXE and AT-EXE groups. There were no differences in age, BMI, or handedness between AU-EXE and AT-EXE groups. Independent one-tailed t-tests were performed to compare VO_2_peak and Wpeak between AU-EXE and AT-EXE groups. The AU-EXE group had significantly lower VO_2_peak and Wpeak than the AT-EXE group.

**Table 1:** Demographic characteristics.

Variable	REST	EXE	t-test (REST vs. EXE)
**REST versus EXE**			
N (Sex)	22 (10 F, 12 M)	22 (11 F, 11 M)	–
Age (years)	24 ± 3	23 ± 2	*t*(42) = − 0.85, *P* = 0.40, *d* = 0.26
BMI (kg m^−2^)	22 ± 3	23 ± 3	*t*(42) = 1.06, *P* = 0.30, *d* = 0.32
Handedness (%)	84 ± 17	89 ± 10	*t*(42) = 1.20, *P* = 0.24, *d* = 0.36
VO_2_peak (ml kg^−1^ min^−1^)	41.0 ± 12.4	40.7 ± 11.7	*t*(42) = − 0.09, *P* = 0.93, *d* = 0.28
Wpeak	262 ± 85	270 ± 87	*t*(42) = 0.31, *P* = 0.76, *d* = 0.09

Variable	AU-EXE	AT-EXE	t-test (AU-EXE vs. AT-EXE)
**AU-EXE versus AT-EXE**			
N (Sex)	12 (6 F, 6 M)	10 (5 F, 5 M)	–
Age (years)	23 ± 2	24 ± 3	*t*(20) = − 1,21, *P* = 0.24, *d* = 0.51
BMI (kg m^−2^)	23.1 ± 3.3	23.6 ± 1.7	*t*(20) = − 0.37, *P* = 0.72, *d* = 0.16
Handedness (%)	92 ± 11	87 ± 8	*t*(20) = 1.24, *P* = 0.23, *d* = 0.53
VO_2_peak (ml kg^−1^ min^−1^)	31.6 ± 4.2	51.5 ± 7.6	*t*(20) = − 7.74, ***P*** **=** **0.001**, *d* = 1.70
Wpeak	205 ± 47	348 ± 53	*t*(20) = − 6.68, ***P*** **=** **0.001**, *d* = 1.63

Values are reported as mean ± SD (see Supplementary Table S2 online for sex-specific data). Significant *P*-values (< 0.05) are bolded.

### Motor skill acquisition

During Visit 2, participants practiced a visuomotor tracking task for six blocks (B1-6), where each block consisted of 20 trials (‘motor skill acquisition’). Performance was evaluated by Time on Target (ToT), a measure of how successful participants were in tracking a series of on-screen targets using a cursor.

**Figure 2:**
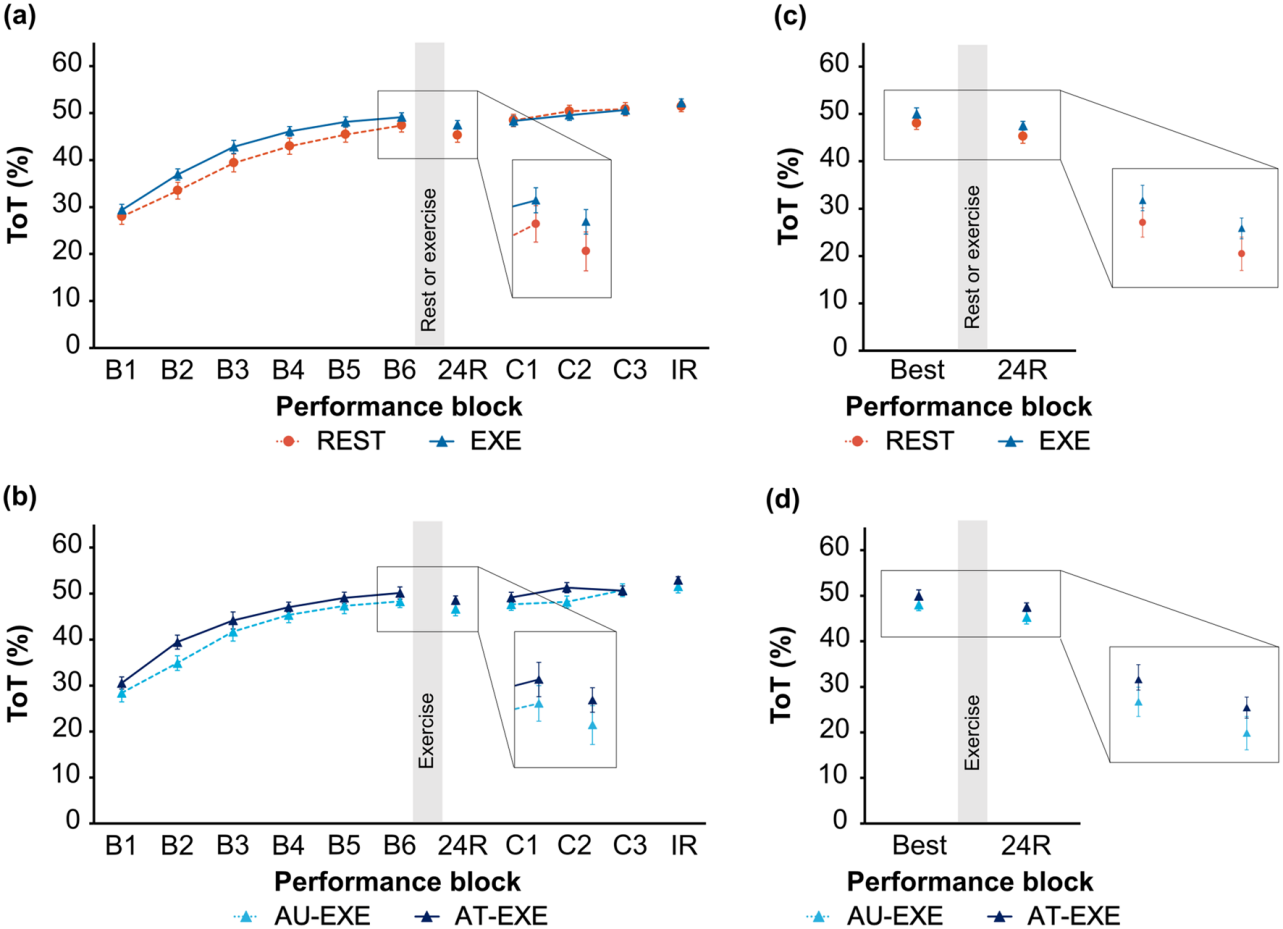
Motor skill performance curves for REST versus EXE groups, and AU-EXE versus AT-EXE groups. Mean Time on Target (ToT) scores (20 trials per block) for acquisition (B1-6), 24-h retention test (24R), ceiling test (C1-3), and immediate retention test (IR) are plotted for (**a**) REST and EXE groups, and (**b**) AU- EXE and AT-EXE groups. Participants rested or exercised (shown as grey bar) immediately after B6. Mean ToT scores for participant’s best block of acquisition (Best) and 24-h retention test (24R) are plotted for (**c**) REST and EXE groups, and (**d**) AU-EXE and AT-EXE groups. All data represent mean ± SEM.

To address the primary objective, we performed a 2 × 6 mixed ANOVA with between-subjects factor condition (REST, EXE) and within-subjects factor time (B1-6). There was a significant main effect of time, where REST and EXE groups significantly improved their ToT across the six blocks of motor skill acquisition (Fig. 2a). There was no significant main effect of condition or two-way interaction (see Table 2 for full reporting of statistical results). REST and EXE groups did not differ in baseline (B1) motor skill performance, as indicated by an independent two-tailed t-test (Table 2).

**Table 2:** Results of motor skill performance statistical analyses.

ANOVA	Baseline (B1)	Acquisition (B1-6)	Retention (Best, 24R)	Ceiling (24R, IR)
**REST versus EXE**				
Condition (REST, EXE)	*t*(42) = − 0.69, *P* = 0.50, *d* = 0.21	*F*(1, 42) = 2.03, *P* = 0.16, η_p_^2^ = 0.05	*F*(1, 42) = 1.70, *P* = 0.20, η_p_^2^ = 0.04	*F*(1, 42) = 1.12, *P* = 0.30, η_p_^2^ = 0.03
Time	–	*F*(3.17, 133.22) = 269.13, ***P*** **=** **0.001**, η_p_^2^ = 0.86	*F*(1, 42) = 35.83, ***P*** **=** **0.001**, η_p_^2^ = 0.46	*F*(1, 42) = 80.72, ***P*** **=** **0.001**, η_p_^2^ = 0.66
Condition × time	–	*F*(3.17, 133.22) = 0.90, *P* = 0.45, η_p_^2^ = 0.02	*F*(1, 42) = 0.11, *P* = 0.74, η_p_^2^ = 0.003	*F*(1, 42) = 1.40, *P* = 0.24, η_p_^2^ = 0.03
**AU-EXE versus AT-EXE**				
Fitness level (AU, AT)	*t*(20) = − 0.87, *P* = 0.39, *d* = 0.38	*F*(1, 20) = 1.43, *P* = 0.25, η_p_^2^ = 0.07	*F*(1, 20) = 1.66, *P* = 0.21, η_p_^2^ = 0.08	*F*(1, 20) = 1.00, *P* = 0.33, η_p_^2^ = 0.05
Time	–	*F*(3.07, 61.44) = 155.95, ***P*** **=** **0.001**, η_p_^2^ = 0.89	*F*(1, 20) = 14.61, ***P*** **=** **0.001**, η_p_^2^ = 0.42	*F*(1, 20) = 60.66, ***P*** **=** **0.001**, η_p_^2^ = 0.75
Fitness level × time	–	*F*(3.07, 61.44) = 0.81, *P* = 0.50, η_p_^2^ = 0.04	*F*(1, 20) = 0.20, *P* = 0.66, η_p_^2^ = 0.01	*F*(1, 20) = 0.16, *P* = 0.69, η_p_^2^ = 0.008

Motor skill performance during baseline, acquisition, retention, and ceiling were compared between REST versus EXE groups, and AU-EXE and AT-EXE groups. Significant *P*-values (< 0.05) are bolded.

To address the secondary objective, we performed a 2 × 6 mixed ANOVA with between-subjects factor fitness level (AU, AT) and within-subjects factor time (B1-6). There was a significant main effect of time, where AU-EXE and AT-EXE groups significantly improved their ToT across the six blocks of motor skill acquisition (Fig. 2b). There was no significant main effect of fitness level or two-way interaction (Table 2). AU-EXE and AT-EXE groups did not differ in baseline (B1) motor skill performance, as indicated by an independent two-tailed t-test (Table 2).

### Motor skill retention

During Visit 3, which took place 24 ± 2-h after Visit 2, participants completed a motor skill retention test that involved one block of 20 trials. We followed the approach of Nepveu et al. and compared motor skill performance between the best block of acquisition (Best) and the 24-h retention test (24R)^27^.

To address the primary objective, we performed a 2 × 2 mixed ANOVA with between-subjects factor condition (REST, EXE) and within-subjects factor time (Best, 24R). There was a significant main effect of time, where REST and EXE groups had significantly lower ToT for the 24-h retention test compared to their best block of acquisition (Fig. 2c). There was no significant main effect of condition or two-way interaction (Table 2).

**Figure 3:**
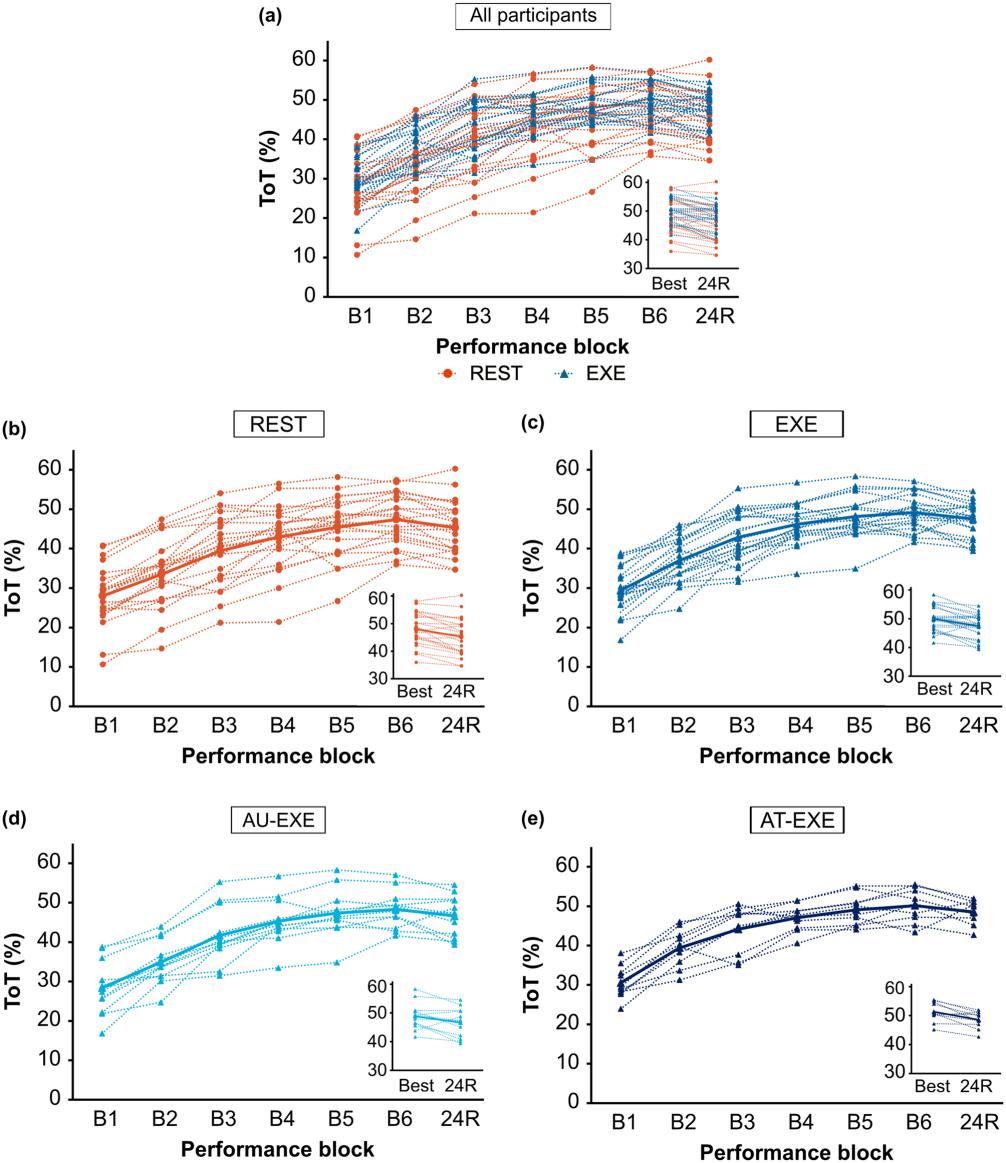
Individual motor skill performance curves. For each participant, mean Time on Target (ToT) scores (20 trials per block) are shown for acquisition (B1-6), best block of acquisition (Best), and 24-h retention test (24R). (**a**) All participants, categorized by REST or EXE group. (**b**–**e**) For each group, dotted lines represent individual data curves and solid lines represent means for each respective group.

Motor skill retention was also evaluated at the individual level for REST and EXE groups by calculating the difference between 24R and Best ToT, commonly referred to as relative retention (Δ ToT) (see Fig. 3a–c for individual performance curves). To characterize Δ ToT, the arbitrary cut off value of zero was used to distinguish between motor skill performance decrements (Δ ToT < 0) and offline consolidation gains (Δ ToT > 0). Thirty-eight out of 44 (86%) participants showed performance decrements, where the magnitude of decrements was relatively small and ranged from 0–9% (*M* = 4%). Six out of 44 (14%) participants showed offline consolidation gains. Among these six participants, three were from the REST group (Δ ToT = 0.13, 1.74, 2.08) and three were from the EXE group (Δ ToT = 1.09, 1.24, 4.91). In addition, when more conservative cut off values were used, 31 out of 44 (~ 70%) participants still showed performance decrements (Δ ToT < − 1), while eight out of 44 (~ 18%) showed performance maintenance (− 1 ≤ Δ ToT ≤ 1), and five out of 44 (~ 11%) showed offline consolidation gains (Δ ToT > 1).

To address the secondary objective, we performed a 2 × 2 mixed ANOVA with between-subjects factor fitness level (AU, AT) and within-subjects factor time (Best, 24R). There was a significant main effect of time, where AU-EXE and AT-EXE groups had significantly lower ToT for the 24-h retention test compared to the best block of acquisition (Fig. 2d). There was no significant main effect of fitness level or two-way interaction (Table 2).

Among AU-EXE and AT-EXE participants, 19 out of 22 (86%) participants showed performance decrements (Δ ToT < 0), and three out of 22 (14%) participants showed offline consolidation gains (Δ ToT > 0) (see Fig. 3d and Fig. 3e for individual performance curves). Among these three participants, two were from the AU-EXE group (Δ ToT = 1.24, 4.91) and one was from the AT-EXE group (Δ ToT = 1.09). When more conservative cut off values were used, 15 out of 22 (68%) participants still showed performance decrements (Δ ToT < − 1), while four out of 22 (18%) showed performance maintenance (− 1 ≤ Δ ToT ≤ 1), and three out of 22 (14%) showed offline consolidation gains (Δ ToT > 1).

### Ceiling

After the 24-h retention test, participants performed an additional three blocks of practice (ceiling test blocks, C1-3) to determine if they were at ceiling in terms of their motor skill performance. After the ceiling test blocks, participants performed one last block of 20 trials, referred to as the immediate retention test (IR).

To test ceiling effects in the REST and EXE groups, we performed a 2 × 2 mixed ANOVA with between-subjects factor condition (REST, EXE) and within-subjects factor time (24R, IR). There was a significant main effect of time, where REST and EXE groups had significantly higher ToT for the immediate retention test compared to the 24-h retention test. There was no significant main effect of condition or two-way interaction (Table 2).

To test ceiling effects in the AU-EXE and AT-EXE groups, we performed a 2 × 2 mixed ANOVA with between-subjects factor fitness level (AU, AT) and within-subjects factor time (24R, IR). There was a significant main effect of time, where AU-EXE and AT-EXE groups had significantly higher ToT for the immediate retention test compared to the 24-h retention test. There was no significant main effect of fitness level or two-way interaction (Table 2).

### High-intensity interval training (HIIT)

The exercise protocol involved 20-min of HIIT, which consisted of 10 × 1-min high-intensity intervals (90% Wpeak) interspersed with 1-min low-intensity intervals (25% Wpeak). All participants completed the exercise protocol. However, two AU-EXE participants took breaks (*M* = 50 s, *SD* = 17 s, range = 30–60 s) during the high-intensity intervals (*M* = 2; *SD* = 1, range = 1–2). In addition, nine AU-EXE participants took breaks (*M* = 36 s, *SD* = 16 s, range = 10–55 s) during the low-intensity intervals (*M* = 4, *SD* = 2, range = 4–7). No AT-EXE participants took breaks during the high- or low-intensity intervals.

Heart rate (HR), rating of perceived exertion (RPE), and feeling scale (FS) scores were recorded during every minute of the HIIT protocol. Independent two-tailed t-tests were performed to compare HR, RPE, and FS scores between AU-EXE and AT-EXE groups (Table 3). Maximum HR achieved during HIIT, average HR achieved during HIIT, average HR achieved during high-intensity intervals, and average RPE achieved during low-intensity intervals were higher in the AU-EXE group compared to the AT-EXE group.

**Table 3:** HIIT data for AU-EXE and AT-EXE groups.

	AU-EXE	AT-EXE	t-test (AU-EXE vs. AT-EXE)
**HR (% HRpeak)**			
HIIT: maximum HR	101 ± 3	95 ± 4	*t*(20) = 3.86, ***P*** **=** **0.001**, *d* = 1.28
HIIT: average HR	87 ± 4	84 ± 4	*t*(20) = 2.21, ***P*** **=** **0.04**, *d* = 0.87
High-intensity: average HR	86 ± 3	81 ± 4	*t*(20) = 2.99, ***P*** **=** **0.007**, *d* = 1.09
Low-intensity: average HR	90 ± 4	87 ± 4	*t*(20) = 1.55, *P* = 0.14, *d* = 0.64
**RPE score**			
HIIT: maximum RPE	9 ± 1	8 ± 1	*t*(20) = 0.93, *P* = 0.36, *d* = 0.40
High-intensity: average RPE	6 ± 1	6 ± 1	*t*(20) = − 0.30, *P* = 0.77, *d* = 0.13
Low-intensity: average RPE	5 ± 1	4 ± 1	*t*(20) = 3.02, ***P*** **=** **0.007**, *d* = 1.10
**FS score**			
High-intensity: average FS	2 ± 2	3 ± 2	*t*(20) = − 1.28, *P* = 0.22, *d* = 0.54
Low-intensity: average FS	2 ± 2	3 ± 1	*t*(20) = − 1.59, *P* = 0.13, *d* = 0.66

Values are reported as mean ± SD. Significant *P*-values (< 0.05) are bolded. HR, Heart Rate; HRpeak, peak HR achieved during graded exercise test; HIIT, high-intensity interval training; High-intensity, high-intensity intervals during HIIT; Low-intensity, low-intensity intervals during HIIT; RPE, rating of perceived exertion, scores range from 0 (rest) to 10 (maximal); FS, feeling scale, scores range from − 5 (very bad) to + 5 (very good); see Supplementary Table S3 online for sex-specific data and HR data reported in beats per minute.

### Exploratory analyses

To further explore the relationship between aerobic fitness level and motor skill performance, Pearson correlation analyses were conducted between VO_2_peak and three motor skill performance variables: (a) relative retention (Δ ToT), (b) total amount of acquisition (difference in ToT between Best and B1), and c) baseline (B1) acquisition performance. There were no significant correlations between VO_2_peak and relative retention (*r* = 0.03, *P* = 0.90), VO_2_peak and total amount of acquisition (*r* = 0.05, *P* = 0.76), or VO_2_peak and baseline acquisition performance (*r* = 0.09, *P* = 0.55) (see Supplementary Fig. S1 online for scatterplots).

## Discussion

The aims of this study were to determine: (a) if a single session of aerobic exercise enhances motor skill retention, and (b) if aerobic fitness level affects motor skill retention after a single session of aerobic exercise. We found that aerobic exercise did not enhance motor skill retention. We also found no differences in motor skill retention between Aerobically Untrained and Aerobically Trained individuals when their motor skill acquisition was immediately followed by aerobic exercise.

Regardless of whether participants exercised or rested, there were small decrements in motor skill performance at the 24-h retention test relative to the best block of acquisition. These decrements were small, but consistent across participants, with 38 out of 44 (86%) showing this “forgetting”. Six out of 44 (14%) participants showed offline consolidation gains, but the magnitude of these gains was small. Some motor memories do not result in offline consolidation gains and are simply maintained^28^. However, one could also argue offline consolidation gains may have been dampened if participants reached their motor skill performance ceiling at the end of acquisition^1^. But our participants continued improving their motor skill performance across the ceiling test blocks, and thus, were not at motor skill performance ceiling. In sum, we did not replicate past studies that found aerobic exercise enhanced motor skill retention.

A recent meta-analysis found a small, positive effect (Cohen’s *d* = 0.30) of a single session of aerobic exercise on motor memory consolidation^13^. Among studies that have found positive effects of exercise^4,7,8,10–12^, their sample sizes (n = 10–20 per group) were comparable to our study (n = 22 per group). Therefore, the relatively small effect of aerobic exercise on motor skill retention may have been washed out in our study due to the variations in our exercise protocol and motor task parameters compared to previous studies.

The exercise protocol we selected may explain why we did not replicate earlier studies. Four studies have demonstrated enhancements in motor skill retention when high-intensity aerobic exercise was performed after motor skill acquisition^4,7,11,12^. These studies used a 15-min HIIT cycling protocol, which involved three 3-min high-intensity intervals (90% Wpeak) alternated with three 2-min low-intensity intervals^4,7,11,12^. However, recent work from our lab revealed that some individuals with low aerobic fitness levels were unable to complete the entire 15-min HIIT protocol due to volitional exhaustion^29^. Therefore, our study used an alternative HIIT cycling protocol that has successfully been used in sedentary^30,31^ and recreationally active healthy young adults^32^. Our protocol was 20-min and involved 10 × 1-min high-intensity intervals (90% Wpeak) alternated with 10 × 1-min low-intensity intervals (25% Wpeak). This alternative protocol was chosen since it’s shorter 1-min high-intensity intervals may be easier for AU individuals to complete compared to the longer 3-min high-intensity intervals from the previous protocol.

The effect of aerobic exercise on motor skill retention is intensity dependent^12^. Exercise protocols with varying intensity levels and interval durations may lead to different heart rate and metabolic responses in participants. For example, the EXE group achieved lower heart rates compared to previous studies using the 3-min high-intensity/2-min low-intensity protocols^11,12^. This discrepancy in heart rate also existed when only comparing the AT-EXE group to past studies using the 3-min high-intensity/2-min low-intensity protocols. Moreover, two studies using moderate-intensity aerobic exercise protocols did not observe an effect of exercise on motor skill retention^12,15^. The heart rates achieved by these moderate-intensity aerobic exercise protocols were lower than the heart rates achieved during our protocol^12,15^. Therefore, our exercise protocol and the moderate-intensity aerobic exercise protocols may not have induced enough cardiovascular challenge to yield exercise enhancements in motor skill retention.

Furthermore, our study’s high-intensity intervals may have been too short to strain the anaerobic metabolic system and induce sufficient release of lactate. Previous studies have demonstrated that exercise protocols with shorter high-intensity interval durations result in lower blood lactate concentrations after exercise^33^. Lactate may be an important metabolic component for several reasons. Firstly, higher blood lactate concentrations after a single session of aerobic exercise have been associated with superior motor skill retention^8^. Secondly, lactate may regulate other biomarkers that could be involved in enhancing motor learning. For example, post-exercise blood lactate concentrations have been positively associated with post-exercise CSE changes^34^ and post-exercise BDNF concentrations^35^; though a second study did not find the same association with BDNF^8^. Lastly, lactate may support long-term memory formation^36^. Therefore, if our HIIT protocol was optimized for interval duration and intensity, the EXE group may have had a stronger lactate response and perhaps superior motor skill retention than the REST group. In addition, performing exercise at higher intensities has been associated with greater post-exercise lactate concentrations^12^. Although our exercise protocol’s intensities were standardized relative to each individual’s peak workload, the AT-EXE group still exercised at higher intensities, on an absolute level, compared to the AU-EXE group. Therefore, with an optimized HIIT protocol, the AT-EXE group may have had a greater lactate response than the AU-EXE group, and perhaps superior motor skill retention.

Differences in our motor task parameters compared with past studies may also explain why we did not replicate previous findings. Our study’s motor task was conceptually similar to four studies that found an effect of exercise on motor skill retention^4,7,11,12^, such that all five studies used continuous, explicit visuomotor tracking tasks. We cannot rule out that differences in details across the tasks may have contributed to our lack of replication. However, more importantly, differences in our findings may be related to how we calculated motor skill retention. Specifically, Dal Maso et al. had participants practice a visuomotor tracking task (acquisition), followed by exercise, and two retention tests (8-h and 24-h after acquisition)^4^. In contrast, our study did not include an 8-h retention test. Participants in the Dal Maso study had decrements in performance during the 8-h retention test relative to the end of motor skill acquisition. Therefore, when 24-h motor skill retention was compared with 8-h motor skill retention, it used a less conservative baseline for comparison as opposed to the end of acquisition. Furthermore, the Dal Maso et al. 8-h and 24-h retention tests had 40 trials each. In contrast, our study’s 24-h retention test only had 20 trials. The extra 20 trials during their 24-h retention test may have led to a practice effect, and thus, better motor skill performance during the 24-h retention test.

Three other parameter modifications in our motor task compared to Dal Maso et al.^4^ may have also contributed to differences in our findings. First, our motor task provided online feedback of the cursor’s full tail during trials (i.e. the entire path the cursor travelled through was visible; see Fig. 5b in “Methods” section), while theirs only showed a short portion of the cursor’s tail during trials. Since online feedback is known to help facilitate learning^1^, our task may have been less challenging. Second, our motor task only included five familiarization trials prior to acquisition, while theirs involved participants training to the criterion score of 30 before acquisition. Despite these two differences, the motor skill acquisition performance curves between the studies appear to follow similar trajectories. Lastly, our motor task involved producing a pinching force (thumb and index finger) to control the cursor, while theirs involved producing a grasping force (entire hand). Therefore, our motor task involved more fine motor skills. However, based on the theory of generalized motor programs, one would expect generalizability between two similar motor tasks using different effectors^37^. Therefore, while these factors could have additionally contributed to the differences in our findings, they are unlikely to be leading explanations.

The general effect of aerobic exercise on motor learning appears to be unclear. Some studies have demonstrated an effect of aerobic exercise on motor skill acquisition^5,9^, motor skill retention^4,7,8,10–12^, and motor skill retention retrieval^6^. In contrast, other studies have not demonstrated an effect of aerobic exercise on motor learning^15,17,38^. This may mean that there is a beneficial effect of aerobic exercise, but the effects are specific to the participants tested, and the exercise and motor task parameters used. Furthermore, there may be individual differences with respect to the metabolic and neural responses that underlie exercise-induced enhancements in motor learning. For example, biomarkers like BDNF and CSE have been suggested to mediate the effects of aerobic exercise on motor learning. However, variability in BDNF and CSE responses to aerobic exercise have also been reported. A recent meta-analysis found a medium effect of BDNF concentration increases after a single session of aerobic exercise^24^. But the effect was inconsistent and heterogenous, with 61% of the studies reporting no significant change in BDNF after aerobic exercise^24^. Considerable variability in exercise-induced changes in CSE has also been observed in both sedentary^23,39^ and active individuals^23^, with a spread of individuals showing increases, decreases, and no changes in CSE after aerobic exercise. Further investigation of such biomarkers may be helpful to understand exercise effects on motor learning.

Future studies should investigate the impact of long-term aerobic exercise training (i.e. weeks to months) paired together with long-term motor learning, which may yield greater enhancements in motor learning than a single session of exercise. This would supplement recent findings that long-term aerobic exercise (seven training sessions over two weeks) enhanced subsequent motor learning on a novel balance task over a period of six weeks^40^. Future research should also explore motor tasks with more real world relevance. Studies in the field of exercise and motor learning have mainly used laboratory-based tasks. While these tasks are useful due to the high amount of control and relative task simplicity, they may not generalize to more complex, real-world contexts^41,42^. More complex, real-world motor tasks (e.g. tasks using more effectors or multi-joint movements, such as playing the piano) may allow for greater challenge, and thus, a greater window of opportunity for exercise enhancements^43^. In addition, future research should investigate clinical populations who may also have a greater window of opportunity for exercise enhancements. Specifically, studies should further examine how exercise can be used as a motor rehabilitation strategy after neural injury. This would complement preliminary findings that exercise may help promote poststroke motor relearning^27^, functional recovery^44^, and neuroplasticity^45^.

In conclusion, this study examined the effect of a single session of aerobic exercise versus rest on motor skill retention, and explored the effect of aerobic fitness level on motor skill retention after exercise. We found that aerobic exercise did not enhance motor skill retention. Furthermore, we found that aerobic fitness level did not modify motor skill retention after exercise. Discrepancies in our findings compared to previous literature may be due to the variations in our exercise and motor task parameters compared to previous studies.

## Methods

### Participants

A sample of convenience was recruited from the University of Toronto and the Greater Toronto Area using advertisements posted on campus and email lists for University of Toronto students. Participants were screened for eligibility: (i) age 20–29, (ii) right-handed, according to the Edinburgh Handedness Inventory^46^, (iii) no history of neurological or psychiatric diagnoses, (iv) no intake of medications affecting the central nervous system, (v) no consumption of nicotine or cannabis, (vi) no competitive videogaming experience, (vii) no cardiorespiratory, musculoskeletal, pulmonary, or hormone-related conditions.

Eligible participants were invited for aerobic fitness level screening. Participants were required to achieve a VO_2_peak within the bottom 25% (Aerobically Untrained; female: ≤ 33.0 ml kg^−1^ min^−1^; male: ≤ 39.0 ml kg^−1^ min^−1^) or top 25% (Aerobically Trained; female: ≥ 42.4 ml kg^−1^ min^−1^; male: ≥ 48.5 ml kg^−1^ min^−1^) of age- and sex-specific normative values^16^. The study protocol was approved by the University of Toronto Health Sciences Research Ethics Board (REB # 36226) and all methods were performed in accordance with relevant guidelines and regulations. All participants provided written, informed consent before any experimental procedures began.

Due to limitations of a small sample size, sex-based statistical analyses were not conducted. However, sex-specific data are reported in Supplementary Tables S2 and S3.

### Experimental study design

**Figure 4:**
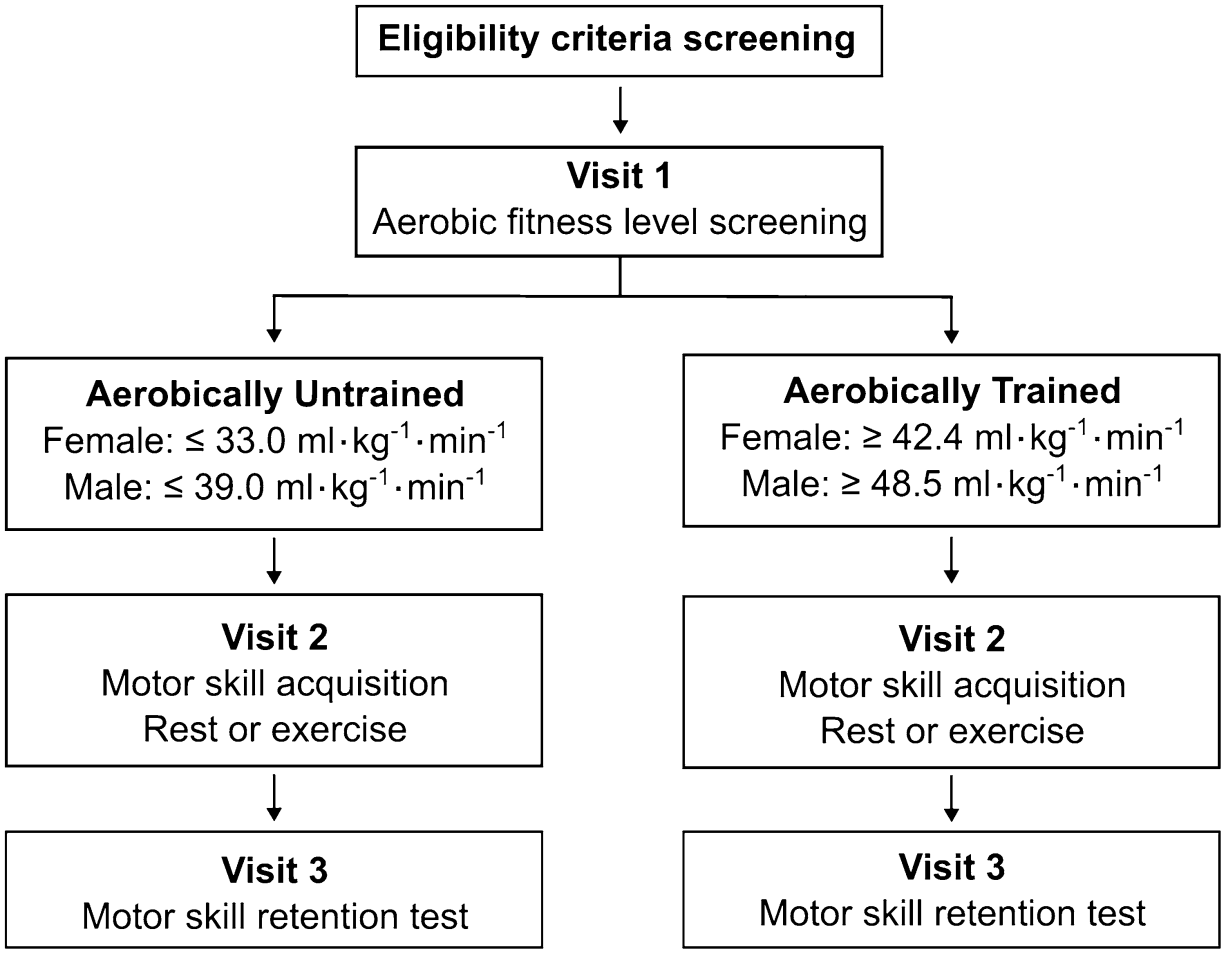
Study design schematic.

The study included three visits—Visit 1: aerobic fitness level screening; Visit 2: motor skill acquisition, followed by either rest (REST) or exercise (EXE); Visit 3: motor skill retention test (Fig. 4). Participants who met the aerobic fitness level criteria were assigned to the Aerobically Untrained (AU) or Aerobically Trained (AT) group, then pseudorandomized into the REST or EXE group to match age and sex.

Participants were instructed to avoid vigorous physical activity: (a) 24-h before Visits 1 and 2 to minimize interference on exercise performance, and (b) 2-h after Visit 2 and 2-h before Visit 3 to minimize interference on motor skill performance^11^. Participants were instructed to avoid caffeine 2-h before Visits 1–3 and 2-h after Visit 2. To minimize caffeine withdrawal effects, efforts were made to schedule visits at least 2-h after regular caffeine intake times.

### Visit 1: aerobic fitness level screening

To determine VO_2_peak, participants completed a maximal graded exercise test (GXT) on a cycle ergometer (Ergomedic 839E, Monark, Sweden). Oxygen consumption, expired carbon dioxide, pulmonary ventilation, and respiratory exchange ratio (RER) were monitored via a metabolic cart (ParvoMedics TrueOne 2400, Sandy, UT, USA). Heart rate (HR) was measured via telemetry (Polar H7). After a 2-min warm up (50 W; self-selected cadence), the GXT workload increased by 1 W every two seconds [60–90 revolutions per minute (rpm)] until volitional exhaustion or inability to maintain a cadence of 60 rpm.

To confirm VO_2_peak was reached, at least two of the following criteria were required^16^: (a) VO_2_plateau (< 0.15 L min^−1^), (b) RER > 1.15, and (c) HRpeak within 10 beats of age-predicted maximum heart rate (HRmax)^47^. Eleven participants (six AU, five AT) achieved VO_2_peak values within accepted ranges, but did not meet at least two of the criteria. Therefore, they completed a second GXT. Six participants (four AU, two AT) still did not meet at least two criteria, but were allowed to complete the study since their VO_2_peak, VO_2_plateau, RER, and HRpeak values were similar between the GXTs.

Peak cycling power (Wpeak) achieved during the GXT was used to standardize the exercise protocol during Visit 2. For participants that completed two GXTs, their second Wpeak was used.

### Visit 2: motor skill acquisition, followed by rest or exercise

**Figure 5:**
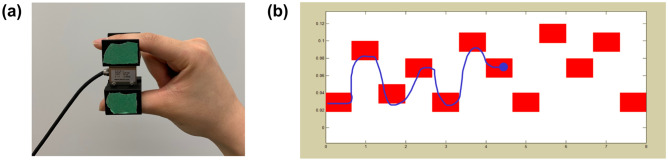
Visuomotor tracking task practiced during acquisition. (**a**) Force transducer (**b**) Participants traced a blue cursor through red targets on a computer screen as accurately as possible. Online feedback of the cursor’s real-time position and tail (i.e. the path the cursor travelled through) were visible during each trial.

For the visuomotor tracking task, participants sat in front of a computer screen and held a strain gauge-based force transducer (DACELL UU3-K50) between their right thumb and index finger (Fig. 5a). The force transducer controlled an on-screen cursor, which automatically moved from left to right (8-s per trial). Participants pinched the force transducer to control the cursor’s vertical movement and maximize the amount of time spent within a series of targets^27^ (Fig. 5b). After each trial, participants were provided a knowledge of results feedback score, referred to as Time on Target (ToT; percentage of time the cursor was inside a target during a trial; maximum score of 100). ToT was used as the measure of motor skill performance on the visuomotor tracking task. The force required to reach the highest target was standardized to 15% of each participant’s maximum voluntary contraction on the force transducer. Participants completed five familiarization trials, then practiced for six blocks of 20 trials (motor skill acquisition) with 2-min of rest between each block.

After motor skill acquisition, participants either exercised or rested for 25-min. The exercise protocol was conducted on the cycle ergometer and exercise intensities were standardized for each participant, as a percentage of their Wpeak. The protocol included a 3-min warm up (25% Wpeak; self-selected cadence), followed by 20-min of HIIT [10 rounds of alternating 1-min high-intensity (90% Wpeak; 85–100 rpm) and 1-min low-intensity (25% Wpeak; self-selected cadence)], and a 2-min cool down (0 W). During every minute of HIIT, participants rated their perceived exertion and affective valence using a modified Borg CR10 Scale^48^ and Feeling Scale^49^. Heart rate was monitored throughout the protocol. During rest, participants silently read magazines (Toronto Life: The City’s 100 Best Restaurants; Ultimate Travel Bucket List) while seated.

### Visit 3: motor skill retention test, ceiling test, immediate retention test

The following day (24 ± 2-h later), participants completed a 24-h retention test (24R; one block of 20 trials) without ToT feedback, as knowledge of results may facilitate learning. Subsequently, participants completed three ceiling test blocks (C1-3; 20 trials per block) with ToT feedback, and an immediate retention test (IR; one block of 20 trials) without ToT feedback. Since IR and 24R did not provide ToT feedback, they could be ‘equally’ compared to evaluate if participants reached a motor skill performance ceiling.

### Statistical analyses

Two sets of analyses were conducted. We first compared REST and EXE groups to evaluate the effect of condition, then performed sub-analyses to compare AU-EXE and AT-EXE groups and explore the effect of fitness level. Normality was confirmed using Shapiro–Wilk tests. If normality was violated, then histograms and Q-Q plots were evaluated. Equal variance and sphericity were confirmed with Levene’s tests and Mauchly’s tests respectively. If sphericity was violated, Greenhouse–Geisser corrections were used. Unless otherwise stated, the chosen alpha level was 0.05.

#### Demographics

To compare mean age, BMI, handedness, VO_2_peak, and Wpeak between REST and EXE groups, independent two-tailed t-tests were used. To compare mean age, BMI, and handedness between AU-EXE and AT-EXE groups, independent two-tailed t-tests were used. To compare VO_2_peak and Wpeak between AU-EXE and AT-EXE groups, independent one-tailed t-tests were used.

#### Motor skill acquisition

Motor skill acquisition mean ToT was compared between REST and EXE groups with a 2 (condition: REST, EXE) × 6 (time: B1-6) mixed ANOVA. Baseline (B1) motor skill acquisition mean ToT was compared between EXE and REST groups with an independent two-tailed t-test. Motor skill acquisition mean ToT was compared between AU-EXE and AT-EXE groups using a 2 (fitness level: AU, AT) × 6 (time: B1-6) mixed ANOVA. Baseline (B1) motor skill acquisition mean ToT was compared between AU-EXE and AT-EXE groups with an independent two-tailed t-test.

#### Motor skill retention

Motor skill retention was compared between REST and EXE groups with a 2 (condition: REST, EXE) × 2 (time: Best, 24R) mixed ANOVA. Motor skill retention was also evaluated at the individual level for REST and EXE groups by comparing each participant’s performance at 24R relative to Best (Δ ToT = 24R mean ToT minus Best mean ToT). The arbitrary cut off value of 0 was used to characterize motor skill performance decrements (Δ ToT < 0) and offline consolidation gains (Δ ToT > 0). Motor skill retention was compared between AU-EXE and AT-EXE groups with a 2 (fitness level: AU, AT) × 2 (time: Best, 24R) mixed ANOVA. Motor skill retention was also evaluated at the individual level for AU-EXE and AT-EXE groups using Δ ToT. The arbitrary cut off value of 0 was used to characterize motor skill performance decrements (Δ ToT < 0) and offline consolidation gains (Δ ToT > 0).

#### Ceiling

To determine if REST and EXE groups reached a motor skill performance ceiling, a 2 (condition: REST, EXE) × 2 (time: 24R, IR) mixed ANOVA was used. To determine if AU-EXE and AT-EXE groups reached a motor skill performance ceiling, a 2 (fitness level: AU, AT) × 2 (time: 24R, IR) mixed ANOVA was used.

#### HIIT

Three categories of variables were analyzed: HR, rating of perceived exertion (RPE), and Feeling Scale (FS). Four HR variables were analyzed: (a) maximum HR during HIIT, (b) average HR during HIIT, (c) average HR during high-intensity intervals, and (d) average HR during low-intensity intervals. Three RPE variables were analyzed: (a) maximum RPE during HIIT, (b) average RPE during high-intensity intervals, and (c) average RPE during low-intensity intervals. Two FS variables were analyzed: (a) average FS score during high-intensity intervals, and (b) average FS score during low-intensity intervals. Independent two-tailed t-tests were used to compare means between AU-EXE and AT-EXE groups.

#### Exploratory analyses

Pearson correlations were performed between VO_2_peak and three motor skill performance variables: (a) relative retention (Δ ToT), (b) total amount of acquisition, (difference between Best and B1 mean ToT), (c) baseline (B1) acquisition performance.

## Supplementary Information

Supplementary Information 1.

Supplementary Information 2.

## Data Availability

The datasets generated and analyzed during this study are included in this published article (see Supplementary Dataset).
