# Supplementary material for: Aerobic exercise and aerobic fitness level do not modify motor learning
Source: Sci Rep. 2021 Mar 8;11:5366. doi: 10.1038/s41598-021-84764-y (PMC7970889; doi:10.1038/s41598-021-84764-y)
Supplement: Supplementary file 1 — Supplementary Information 1. [file 41598_2021_84764_MOESM1_ESM.pdf]

Supplementary Information

Aerobic exercise and aerobic fitness level do not modify motor learning

Andrea Hung[1,2], Marc Roig[3,4], Jenna B. Gillen[1], Catherine M. Sabiston[1], Walter Swardfager[5,6], Joyce L. Chen*[1,2,5]

[1] Faculty of Kinesiology & Physical Education, University of Toronto, Toronto, ON, Canada

[2] Rehabilitation Sciences Institute, University of Toronto, Toronto, ON, Canada

[3] Memory and Motor Rehabilitation Laboratory (MEMORY-LAB), Feil and Oberfeld Research Centre, Jewish Rehabilitation Hospital, Montreal Center for Interdisciplinary Research in Rehabilitation (CRIR), Laval, QC, Canada

[4] School of Physical & Occupational Therapy, McGill University, Montreal, QC, Canada

[5] Hurvitz Brain Sciences Program, Sunnybrook Research Institute, Toronto, ON, Canada

[6] Department of Pharmacology & Toxicology, University of Toronto, Toronto, ON, Canada

* Corresponding Author:
Joyce L. Chen, PhD
Faculty of Kinesiology & Physical Education, University of Toronto
55 Harbord Street, M5S 2W6, Toronto, ON, Canada
Email: joycelynn.chen@utoronto.ca

Supplementary Table S1. VO_2peak and age from prior studies compared to normative VO_2peak values. VO_2peak (mean ± SD) and age (mean ± SD) from prior studies are reported as sex-specific values (F = female; M = male; F + M = females and males combined). Some studies[1-3] did not test VO_2peak and thus are not included in the table.

Study	VO_2peak ($ml \cdot kg^{-1} \cdot min^{-1}$)	Age
Higher VO_2peak		
Mang et al., 2014[4]	40.8 ± 6.5 (F); 49.9 ± 10.2 (M)	24 ± 4 (F + M)
Mang et al., 2016[5]	43.0 ± 10.1 (F); 43.8 ± 5.2 (M)	26 ± 5 (F + M)
Roig et al., 2012[6]	53.2 (M)	24 (M)
Skriver et al., 2014[7]	53.2 (M)	24 (M)
Snow et al., 2016[8]	43.6 ± 2.7 (F); 47.5 ± 2.3 (M)	26 ± 1 (F + M)
Thomas et al., 2016[9]	50.3 (M)	24 ± 2 (M)
Thomas et al., 2016[10]	50.6 (M)	24 (M)
Thomas et al., 2017[11]	52.1 (M)	25 ± 4 (M)
Lower VO_2peak		
Dal Maso et al., 2017[12]	36.6 (F + M)	25 (F + M)
Ostadan et al., 2016[13]	39.4 (F + M)	23 (F + M)

Supplementary Table S2. Sex-specific demographic characteristics for (a) AU-REST and AU-EXE, (b) AT-REST and AT-EXE. Values are reported as mean ± SD.

(a)

	REST (female)	REST (male)	EXE (female)	EXE (male)
**N**	10	12	11	11
**Age (years)**	24 ± 2	24 ± 3	23 ± 3	23 ± 2
**BMI ($kg \cdot m^{-2}$)**	21.4 ± 1.8	23.2 ± 3.6	22.7 ± 3.2	24.0 ± 2.0
**Handedness (%)**	81 ± 20	88 ± 14	94 ± 7	85 ± 10
**VO_2peak ($ml \cdot kg^{-1} \cdot min^{-1}$)**	35.7 ± 9.4	45.4 ± 13.1	36.7 ± 8.7	44.6 ± 13.3
**Wpeak**	217 ± 60	299 ± 87	232 ± 81	308 ± 80

(b)

	AU-EXE (female)	AU-EXE (male)	AT-EXE (female)	AT-EXE (male)
**N**	6	6	5	5
**Age (years)**	22 ± 2	24 ± 2	24 ± 3	23 ± 2
**BMI ($kg \cdot m^{-2}$)**	21.8 ± 3.6	24.5 ± 2.6	23.8 ± 2.1	23.3 ± 0.8
**Handedness (%)**	96 ± 5	87 ± 13	91 ± 8	82 ± 5
**VO_2peak ($ml \cdot kg^{-1} \cdot min^{-1}$)**	29.5 ± 3.1	33.8 ± 4.3	45.4 ± 2.1	57.6 ± 5.0
**Wpeak**	168 ± 35	242 ± 17	308 ± 32	387 ± 32

Supplementary Table S3. Sex-specific HIIT data for (a) AU-EXE and (b) AT-EXE. Values are reported as mean ± SD.

(a)

	AU-EXE (female)	AU-EXE (male)
**Heart Rate (HR), as a % relative to HRpeak achieved during graded exercise test**		
Entire HIIT protocol: maximum HR	101 ± 3	101 ± 3
Entire HIIT protocol: average HR	88 ± 3	86 ± 4
High-intensity intervals: average HR	87 ± 2	85± 4
Low-intensity intervals: average HR	91 ± 4	89 ± 4
**HR (beats per minute)**		
Entire HIIT protocol: maximum HR	183 ± 6	176 ± 8
Entire HIIT protocol: average HR	159 ± 9	150 ± 10
High-intensity intervals: average HR	157 ± 8	147 ± 10
Low-intensity intervals: average HR	163 ± 10	155 ± 10
**Rating of Perceived Exertion (RPE) score**		
Entire HIIT protocol: maximum RPE	9 ± 0	8 ± 2
High-intensity intervals: average RPE	6 ± 1	6 ± 1
Low-intensity intervals: average RPE	5 ± 1	5 ± 2
**Feeling Scale (FS) score**		
High-intensity intervals: average FS	1 ± 1	3 ± 2
Low-intensity intervals: average FS	1 ± 1	3 ± 2

(b)

	AT-EXE (female)	AT-EXE (male)
**Heart Rate (HR), as a % relative to HRpeak achieved during graded exercise test**		
Entire HIIT protocol: maximum HR	99 ± 2	92 ± 3
Entire HIIT protocol: average HR	87 ± 2	80 ± 3
High-intensity intervals: average HR	84 ± 3	78 ± 3
Low-intensity intervals: average HR	90 ± 1	84 ± 3
**HR (beats per minute)**		
Entire HIIT protocol: maximum HR	182 ± 7	170 ± 10
Entire HIIT protocol: average HR	160 ± 6	150 ± 12
High-intensity intervals: average HR	156 ± 7	143 ± 12
Low-intensity intervals: average HR	167 ± 5	156 ± 12
**Rating of Perceived Exertion (RPE) score**		
Entire HIIT protocol: maximum RPE	8 ± 2	8 ± 1

High-intensity intervals: average RPE	6 ± 1	7 ± 0
Low-intensity intervals: average RPE	3 ± 1	4 ± 1
**Feeling Scale (FS) score**		
High-intensity intervals: average FS	3 ± 2	3 ± 2
Low-intensity intervals: average FS	3 ± 2	3 ± 1

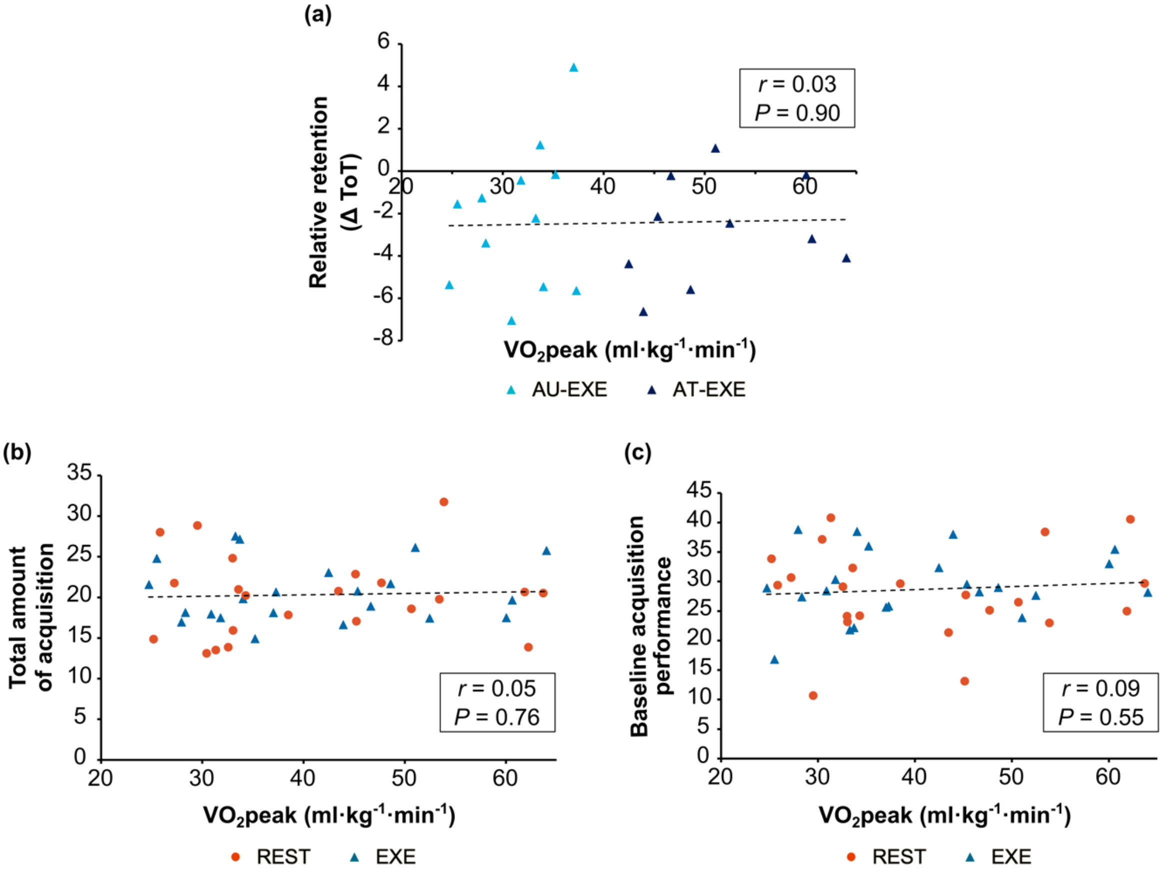

Supplementary Figure S1. Pearson correlations between VO_2peak and motor skill performance measures. (a) VO_2peak and relative retention, **(b)** VO_2peak and total amount of acquisition, **(c)** VO_2peak and baseline acquisition performance.

Supplementary References

1. Statton, M. A., Encarnacion, M., Celnik, P. & Bastian, A.J. A single bout of moderate aerobic exercise improves motor skill acquisition. *PLoS One*. **10,** e0141393; 10.1371/journal.pone.0141393 (2015).
2. Stavrinos, E. L. & Coxon, J. P. High-intensity interval exercise promotes motor cortex disinhibition and early motor skill consolidation. *J. Cogn. Neurosci.* **29,** 593-604 (2017).
3. Lorås, H., Haga, M. & Sigmundsson, H. Effect of a single bout of acute aerobic exercise at moderate-to-vigorous intensities on motor learning, retention and transfer. *Sports (Basel).* **8,** 15; 10.3390/sports8020015 (2020).
4. Mang, C. S., Snow, N. J., Campbell, K. L., Ross, C. J. & Boyd, L. A. A single bout of high-intensity aerobic exercise facilitates response to paired associative stimulation and promotes sequence-specific implicit motor learning. *J. Appl. Physiol. (1985).* **117,** 1325-1336 (2014).
5. Mang, C. S., Snow, N. J., Wadden, K. P., Campbell, K. L. & Boyd, L. A. High-intensity aerobic exercise enhances motor memory retrieval. *Med. & Sci. Sports Exerc.* **48,** 2477-2486 (2016).
6. Roig, M., Skriver, K., Lundbye-Jensen, J., Kiens, B. & Nielsen, J.B. A single bout of exercise improves motor memory. *PLoS One.* **7,** e44594; 10.1371/journal.pone.0044594 (2012).
7. Skriver, K. *et al.* Acute exercise improves motor memory: exploring potential biomarkers. *Neurobiol. Learn. Mem.* **116,** 46-58 (2014).
8. Snow, N. J., Mang, C. S., Roig, M., McDonnell, M. N., Campbell, K. L. & Boyd, L. A. The effect of an acute bout of moderate-intensity aerobic exercise on motor learning of a continuous tracking task. *PLoS One.* **11,** e0150039; 10.1371/journal.pone.0150039 (2016).
9. Thomas, R. *et al.* Acute exercise and motor memory consolidation: the role of exercise timing. *Neural Plast.* **2016,** 6205452; 10.1155/2016/6205452 (2016).
10. Thomas, R. *et al.* Acute exercise and motor memory consolidation: the role of exercise intensity. *PLoS One.* **11,** e0159589; 10.1371/journal.pone.0159589 (2016).
11. Thomas, R. *et al.* Acute exercise and motor memory consolidation: does exercise type play a role? *Scand. J. Med. Sci. Sports*. **27,** 1523-1532 (2017).
12. Dal Maso, F., Desormeau, B., Boudrias, M. H. & Roig, M. Acute cardiovascular exercise promotes functional changes in cortico-motor networks during the early stages of motor memory consolidation. *Neuroimage.* **174,** 380-392 (2018).
13. Ostadan, F., Centeno, C., Daloze, J. F., Frenn, M., Lundbye-Jensen, J. & Roig, M. Changes in corticospinal excitability during consolidation predict acute exercise-induced off-line gains in procedural memory. *Neurobiol. Learn. Mem.* **136,** 196-203 (2016).
